# Mechanisms linking primary biliary cholangitis and osteoporosis: A combined clinical and molecular analysis

**DOI:** 10.1097/MD.0000000000048812

**Published:** 2026-05-22

**Authors:** Hongyu Li, Xinya Guo, Qiaowei Fan

**Affiliations:** a Department of Gastroenterology and Hepatology, The Second Affiliated Hospital of Harbin Medical University, Harbin, Heilongjiang Province, China.

**Keywords:** bioinformatics analysis, biomarker, machine learning, osteoporosis, primary biliary cholangitis

## Abstract

Primary biliary cholangitis (PBC) is an immune-mediated cholestatic liver disease, and osteoporosis (OP) is a prevalent comorbidity that aggravates the disease burden of PBC patients. Although the co-occurrence of the 2 diseases has been widely observed, the underlying molecular mechanisms remain unclear. Herein, we evaluated the causal link between PBC and OP using Mendelian randomization (MR) and explored shared molecular mechanisms through bioinformatics and machine learning. We performed two-sample MR using genome-wide association study summary data for PBC and OP from the Integrative Epidemiology Unit database. Inverse-variance weighting was used as the primary MR method, with heterogeneity and horizontal pleiotropy tests performed to exclude potential biases. To explore shared molecular mechanisms, we analyzed transcriptomic datasets from the Gene Expression Omnibus database, identified comorbidity-associated differentially expressed genes, and applied multiple machine learning algorithms for biomarker screening and validation, combined with immune infiltration analysis. Our study showed that MR demonstrated that PBC significantly increases the risk of OP, while transcriptomic analysis identified 36 shared differentially expressed genes enriched in key biological pathways such as ribonucleic acid splicing and ubiquitin-mediated proteolysis. Furthermore, using 3 machine learning algorithms, we identified 12 PBC-specific and 4 OP-specific diagnostic genes, whose intersection revealed vacuolar protein sorting 37 homolog C (VPS37C) as a common diagnostic biomarker. In both diseases, VPS37C exhibited an area under the receiver operating characteristic curve value >0.7, demonstrating its robust predictive performance. In addition, VPS37C expression was found to be significantly correlated with the infiltration landscape of multiple immune cell types in both PBC and OP. This study identified VPS37C as a shared diagnostic gene linking PBC and OP, providing new insights into their comorbidity at both genetic and immune levels. Our findings further elucidate the molecular mechanisms underlying the comorbidity of PBC and OP, offer novel clues for understanding their pathogenesis, and highlight promising diagnostic and therapeutic targets for clinical application.

## 1. Introduction

Primary biliary cholangitis (PBC) is a chronic immune-mediated cholestatic liver disease characterized by progressive destruction of small intrahepatic bile ducts, mainly affecting middle-aged women.^[1,2]^ Its pathogenesis involves immune dysregulation, abnormal interactions between bile duct epithelial cells (BECs) and immune cells, and disturbances in the gut-liver axis, which drive persistent inflammation and liver fibrosis.^[3]^ Advanced PBC often leads to cirrhosis and extrahepatic complications, including skeletal disorders.^[4–6]^

Osteoporosis (OP) is a prevalent metabolic bone disease featured by decreased bone mass and deteriorated bone microarchitecture. OP is a common and severe complication in PBC patients, with a markedly higher prevalence than in the general population.^[7–9]^ It greatly reduces quality of life and even affects long-term prognosis.

Although a close association between PBC and OP has been widely recognized, the causal relationship and shared molecular mechanisms underlying their comorbidity remain largely unclear. Effective diagnostic biomarkers and targeted strategies for this comorbidity are still lacking.

In recent years, bioinformatics technologies have provided efficient tools for identifying genetic targets that are common across multiple diseases using high-throughput data analysis. This study integrated multi-omics data, including genome-wide association study (GWAS) and high-throughputgene expression profiling data. First, Mendelian randomization (MR) analysis was conducted to evaluate the causal relationship between PBC and OP. Systemic bioinformatics analysis was then conducted to mine disease-specific gene expression patterns. Various machine learning approaches were used to pinpoint disease-associated genes specific to PBC and OP, as well as to identify shared genes between the 2 conditions. In addition, immune infiltration analysis and the construction of a competing endogenous ribonucleic acid (ceRNA) network were employed to further clarify the shared genetic regulatory genes between PBC and OP. This approach provides a theoretical foundation for understanding the molecular mechanisms underlying their comorbidity and identifies potential targets for developing targeted therapeutic agents.

## 2. Materials and methods

### 2.1. Data sources

The GWAS summary statistics were sourced from the open GWAS project of the MRC Integrative Epidemiology Unit database (https://gwas.mrcieu.ac.uk/).^[10]^ The transcriptome analysis datasets used in this study were obtained from the Gene Expression Omnibus database (https://www.ncbi.nlm.nih.gov/geo/). Both the GWAS and transcriptomic data have undergone ethical reviews through their respective original channels and are publicly accessible. Therefore, our study does not require further ethical review.

### 2.2. Exposure and outcome variables in MR analysis

The genetic data pertinent to PBC, identified as ebi-a-GCST90061440, originated from a highly significant international GWAS meta-analysis conducted to date. This study, led by the Cordell team, included 8021 confirmed PBC cases and 16,489 controls of European descent from 5 main European cohorts.^[11]^ A total of 5,004,018 single-nucleotide polymorphism (SNP) loci were analyzed. All confirmed PBC cases met the strict diagnostic criteria set by the European Association for the Study of the Liver, ensuring consistency and reliability in diagnostics. The genetic variable for OP (finn-b-M13_OSTEOPOROSIS) was derived from the Finnish Genome study (FinnGen). This dataset comprised 3203 OP cases and 209,575 controls of Finnish ancestry, analyzing 16,380,452 SNP loci. The OP cases were identified using the International Classification of Diseases, 10th Revision codes. All these data were obtained from the Integrative Epidemiology Unit database, with PBC treated as the exposure and OP as the outcome for further MR analysis.

### 2.3. Selection of instrumental variables

To ensure the reliability of MR analysis and the accuracy of causal inferences, we used the following criteria to screen instrumental variables (IVs): screening strongly associated SNPs: we set a *P*-value threshold of <5 × 10^−8^ to identify SNPs that are strongly associated with the exposure factor. These SNPs were designated as candidate IVs. Excluding SNPs with linkage disequilibrium: we removed SNPs that exhibited linkage disequilibrium based on a distance threshold of 10,000 kb and an *R*^2^ value of 0.001. Eliminating weak IVs via *F*-test: we conducted an *F*-test to select IVs with *F*-test values >10, thereby reducing the impact of weak IVs. Only SNPs that simultaneously meet the above criteria can serve as IVs for subsequent analyses.

### 2.4. MR analysis and sensitivity analysis

MR analysis was conducted using the TwoSampleMR R package. We employed multiple two-sample MR methods to assess the causal effects between PBC and OP, including inverse-variance weighted (IVW), Mendelian randomization-Egger, weighted median, simple mode, and weighted mode. We used IVW as the primary method for MR analysis, while the other 4 methods served as supplementary approaches. In addition, we assessed horizontal pleiotropy using the Egger intercept, evaluated heterogeneity through the Cochran *Q* test, and conducted a sensitivity analysis via leave-one-out analysis. The results were visualized using scatter plots, forest plots, funnel plots, and leave-one-out plots.

### 2.5. Data preprocessing and differentially expressed gene analysis

The raw microarray data of GSE119600 (Illumina HumanHT-12 V4.0 expression beadchip, Illumina Inc.) were downloaded from Gene Expression Omnibus, which contains samples from 90 PBC patients and 47 healthy controls. In addition, we downloaded the GSE56815 microarray data (Affymetrix Human Genome U133A Array, Affymetrix, Inc.), which consists of 40 samples from OP patients and 40 control samples. Differentially expressed genes (DEGs) were identified using the linear models for microarray data R package for correction and analysis, applying an adjusted *P*-value threshold of <.05 for both PBC and OP datasets.^[12]^ The DEGs were visualized using the ggplot2 and pheatmap R packages, which generated volcano plots and heatmaps. To identify the overlapping DEGs between the 2 diseases, we used the ggvenn R package to create a Venn diagram. Finally, the visualization of these intersecting DEGs was accomplished using the circlize R package.

### 2.6. Functional enrichment analysis

We conducted a functional enrichment analysis using the clusterProfiler R package and the Gene Ontology (GO) database.^[13]^ This analysis focused on the intersecting DEGs across 3 categories: biological process (BP), cellular component (CC), and molecular function (MF). In addition, we performed pathway enrichment analysis on these intersecting DEGs based on the Kyoto Encyclopedia of Genes and Genomes (KEGG) biological pathway database. A *P*-value threshold of <.05 was established to identify statistically significant and high-frequency annotations, minimizing background noise.

### 2.7. Machine learning screening for shared feature genes

We utilized the glmnet, e1071, and randomForest R packages to apply least absolute shrinkage and selection operator (LASSO) regression, support vector machine recursive feature elimination (SVM-RFE), and random forest tree methods, respectively.^[14–17]^ Our goal was to identify characteristic genes associated with PBC and OP. The characteristic genes identified by each method were combined, and we took the intersection of these 3 gene sets to obtain disease-specific characteristic genes for both PBC and OP. Finally, we calculated the overlap of the disease-specific genes to identify shared characteristic genes for both conditions. The results were visualized using a Venn diagram.

### 2.8. Protein-protein interaction network of shared feature genes

The protein-protein interaction (PPI) network of shared feature genes was constructed using GeneMANIA (http://genemania.org/).^[18]^ This tool allows us to observe which proteins have regulatory relationships with core genes. A key feature of GeneMANIA is its ability to utilize various association data, including physical interactions, genetic interactions, co-expression, and shared protein domains. This information helps predict and identify potential associated genes based on functional similarity.

### 2.9. Diagnostic value of shared feature genes

Receiver operating characteristic (ROC) curves were generated to evaluate the accuracy of shared genes identified through machine learning. The pROC R package was utilized to plot these ROC curves, with the false positive rate on the x-axis and the true positive rate on the y-axis.^[19]^ The area under the ROC curve (AUC) was calculated, where the AUC value ranges from 0.5 to 1.0. An AUC of 0.7 or higher is generally considered to indicate good discriminatory capability of the model.

### 2.10. Construction of the ceRNA network

We utilized several tools, including miRDB (https://mirdb.org/), miRTarBase (https://awi.cuhk.edu.cn/~miRTarBase/miRTarBase_2025/php/index.php), and TargetScan (https://www.targetscan.org/), to predict the miRNAs that bind to the shared feature genes.^[20–22]^ If all 3 software tools identify a gene as a target of a specific miRNA, we classify it as a target gene for that miRNA. For predicting lncRNAs that interact with miRNAs, we used ENCORI (https://rnasysu.com/encori/).^[23]^ The relationships within the lncRNA-miRNA-mRNA network were then visualized using Cytoscape software (https://cytoscape.org/) to create a ceRNA network diagram.^[24]^ By examining the ceRNA network, we can observe how lncRNAs compete with core genes and their respective relationships with corresponding miRNAs.

### 2.11. Relationships between shared feature genes and immune infiltrating cells

**Figure 1.**
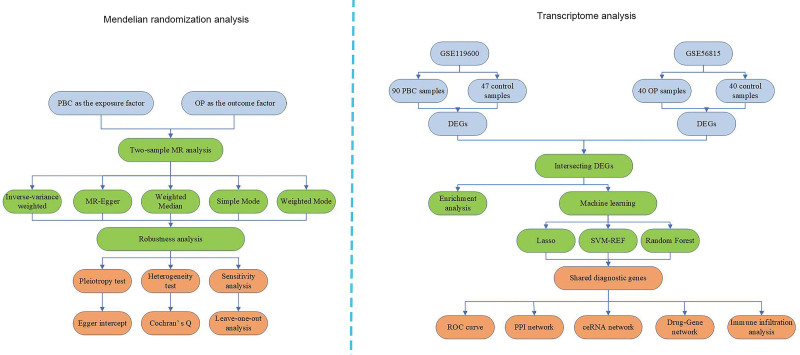
Flowchart of the study.

We utilized the CIBERSORT R package to analyze immune cell infiltration and determine the composition of immune cells in each sample.^[25]^ Samples were filtered based on a *P* value of <.05, retaining only those with higher accuracy. A correlation analysis was conducted between shared feature genes and 22 types of immune cells using the ggplot2 and linkET R packages.^[26]^ The flowchart illustrating this study is presented in Figure 1.

## 3. Results

### 3.1. The causal relationship between PBC and OP

**Figure 2.**
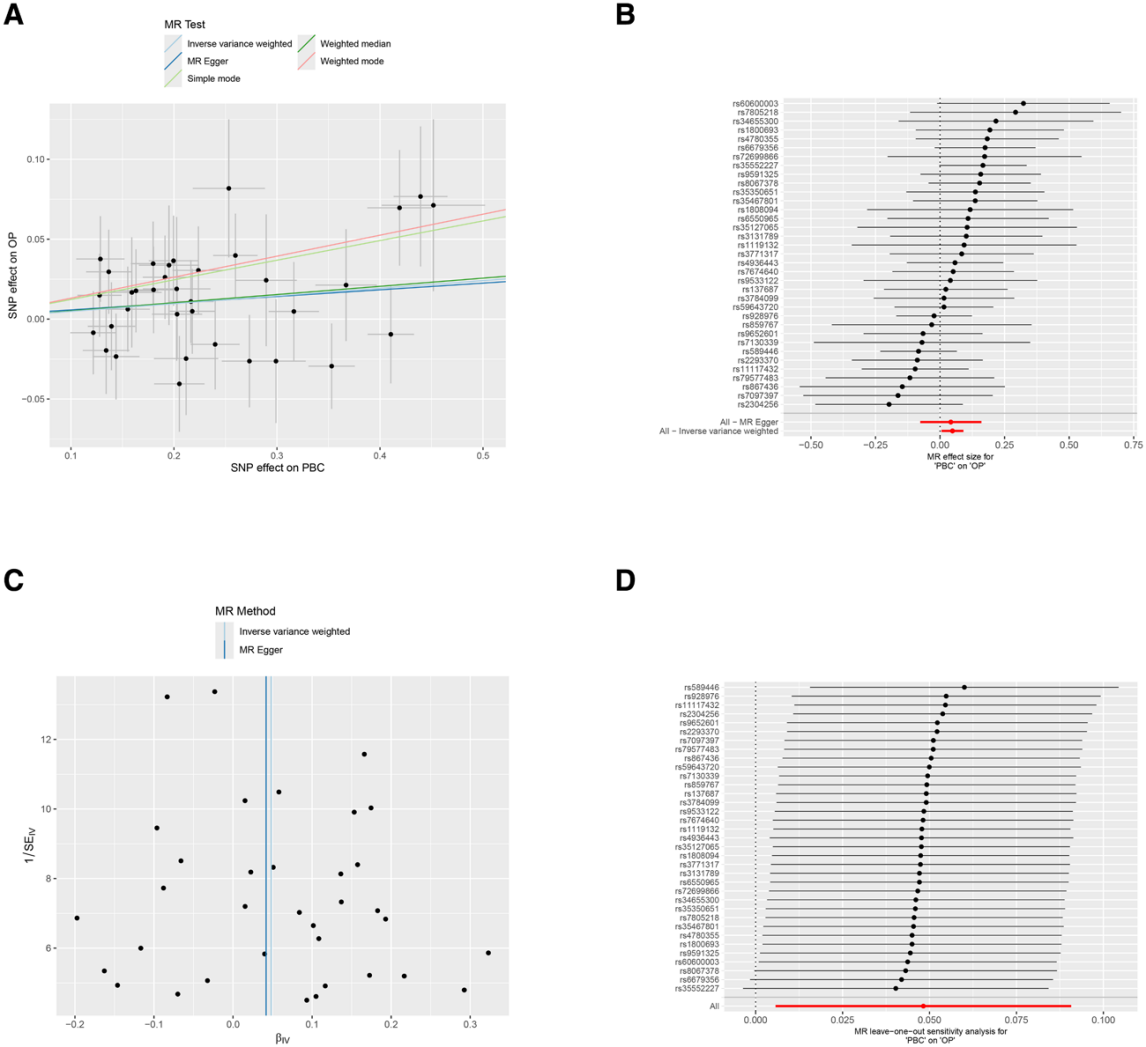
MR analysis illustrating the causal effects of PBC on OP. (A) The scatter plot showing SNPs associated with PBC and their corresponding risk for OP. (B) MR effect size of SNPs associated with PBC and their associated risk for OP. (C) The funnel plot assessing the pleiotropy of the observed causal associations between PBC and OP. (D) The leave-one-out plot of SNPs associated with PBC and their impact on OP. MR = Mendelian randomization, OP = osteoporosis, PBC = primary biliary cholangitis, SNP = single-nucleotide polymorphism.

The results of the MR analysis show a significant positive causal relationship between PBC and OP. Specifically, the onset of PBC is associated with an increased risk of developing OP, as indicated by the following statistics: IVW: *P* = .0262, odds ratio = 1.0494 (95% confidence interval = 1.0057–1.0950; Table 1). In addition, the results of the Egger intercept and Cochran *Q* test produced *P* values >.05, suggesting there is no significant heterogeneity or pleiotropy within the data (Tables S1 and S2). A leave-one-out sensitivity analysis indicated minimal changes in the overall error lines when each SNP was removed individually, with all error lines remaining on the right side of zero. This lack of outliers supports the robustness and reliability of the findings (Fig. 2). Detailed information for specific SNPs used as IVs was summarized in Table S3.

**Table 1:** Results of MR analysis revealing causal relationship between PBC and OP.

Method	*P* value	OR (95% CI)
MR-Egger	.4932	1.0427 (0.9264–1.1736)
Weighted median	.1015	1.0526 (0.9900–1.1192)
Inverse-variance weighted	.0262	1.0494 (1.0057–1.0950)
Simple mode	.1084	1.1307 (0.9771–1.3084)
Weighted mode	.0607	1.1403 (0.9986–1.3019)

CI = confidence interval, MR = Mendelian randomization, OP = osteoporosis, OR = odds ratio, PBC = primary biliary cholangitis.

### 3.2. Identification of intersecting DEGs between PBC and OP

**Figure 3.**
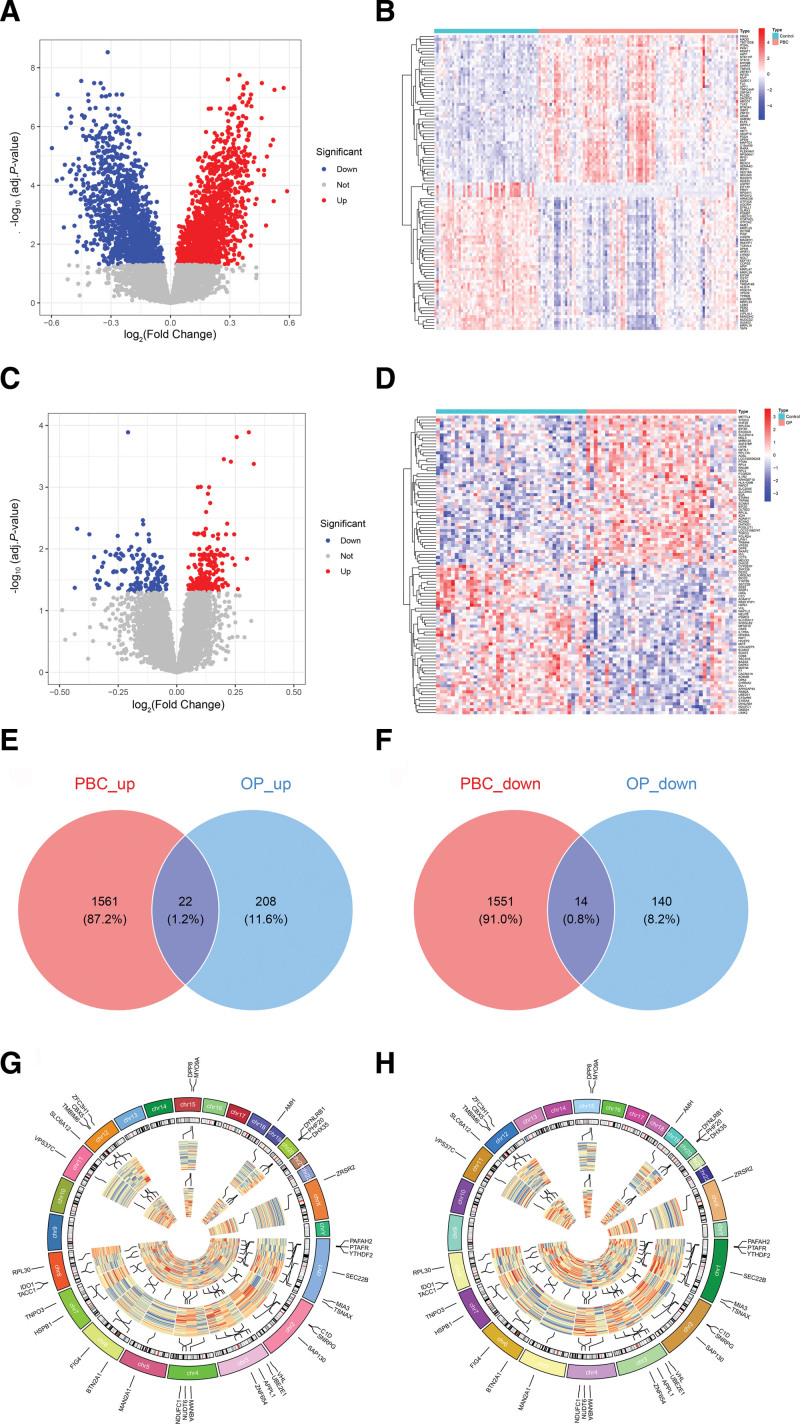
Identification of intersecting DEGs between PBC and OP. (A) Volcano plot of the PBC dataset. (B) Heatmap of the PBC dataset. (C) Volcano plot of the OP dataset. (D) Heatmap of the OP dataset. (E) Upregulated intersecting DEGs. (F) Downregulated intersecting DEGs. (G) Chromosome map of intersecting DEGs in the PBC dataset. (H) Chromosome map of intersecting DEGs in the OP dataset. DEG = differentially expressed gene, OP = osteoporosis, PBC = primary biliary cholangitis. Notes: In volcano plots A and C, the x-axis represents log_2_FC, and the y-axis represents −log10 (adjusted *P* value). The gray dots represent genes that exhibit no differential expression, while the blue dots signify genes that are downregulated. In contrast, the red dots highlight genes that are upregulated. In heatmaps B and D, the x-axis represents samples, while the y-axis represents DEGs. The color coding above the plots indicates the sample groups: bright blue signifies control group samples, and pink denotes disease group samples. The colors within the plots represent gene expression levels, where red indicates high expression and blue indicates low expression. In the chromosome circles labeled G and H, the outermost circle portrays the chromosome numbers, allowing for the labeling of intersecting gene names at their corresponding chromosomal positions. The second circle presents a diagram of the chromosomes, while the third circle displays a heatmap of gene expression in the control group. The fourth circle contains a heatmap of gene expression in the disease group. In these heatmaps, blue indicates low expression and red indicates high expression.

After conducting correction and filtering with the linear models for microarray data package, we identified a total of 3148 DEGs in the PBC dataset. This included 1583 upregulated genes and 1565 downregulated genes (Fig. 3A). In the OP dataset, we identified 384 DEGs, consisting of 230 upregulated genes and 154 downregulated genes (Fig. 3C). Heatmaps illustrating the top 100 most significant DEGs are presented in Figure 3B, D. These DEGs effectively differentiate the disease groups from the control groups. Venn diagrams indicate that there are 36 intersecting DEGs shared between both diseases, including 22 upregulated and 14 downregulated DEGs (as shown in Fig. 3E, F). The chromosomal locations and expression patterns of these intersecting DEGs across both diseases and their respective control groups are illustrated in Figure 3G, H. The list of intersecting DEGs can be found in Table S4.

### 3.3. GO and KEGG enrichment analysis

**Figure 4.**
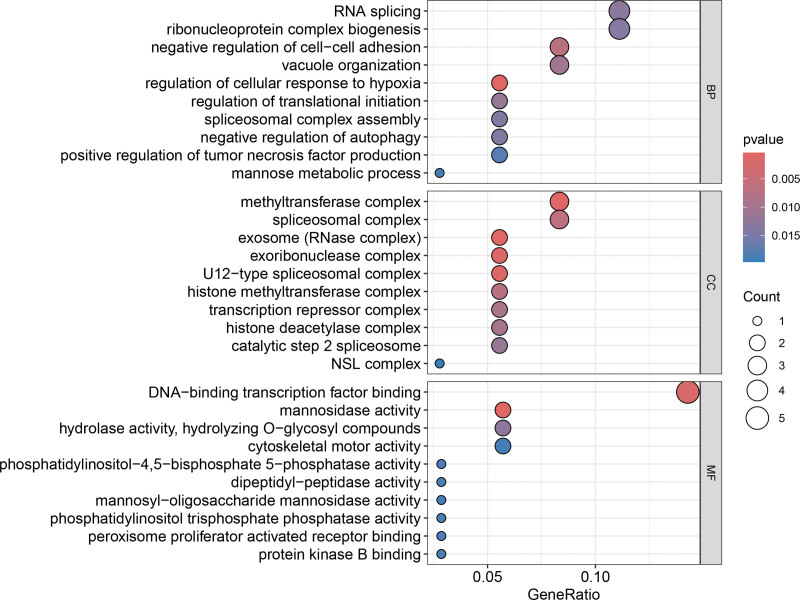
Top 10 significant GO terms enriched by intersecting DEGs in the categories of BP, CC, and MF. BP = biological process, CC = cellular component, DEG = differentially expressed gene, GO = Gene Ontology, MF = molecular function.

By conducting enrichment analysis on the 36 intersecting DEGs, we can identify common physiological and pathological pathways involved in both PBC and OP. The GO enrichment analysis indicates that these intersecting DEGs are primarily involved in BPs such as ribonucleic acid (RNA) splicing, ribonucleoprotein complex biogenesis, regulation of cellular response to hypoxia, vacuole organization, and negative regulation of autophagy. In terms of CCs, these DEGs are mainly associated with methyltransferase complexes, spliceosomal complexes, exosome (RNase complex), and exoribonuclease complexes. Moreover, the enriched MFs include DNA-binding transcription factor binding, cytoskeletal motor activity, hydrolase activity (specifically hydrolyzing O-glycosyl compounds), and dipeptidyl-peptidase activity. The bubble plot of the top 10 significant GO terms enriched by intersecting DEGs in the categories of BP, CC, and MF is displayed in Figure 4. Two KEGG pathways were identified as enriched: hsa04120 (ubiquitin-mediated proteolysis) and hsa00511 (other glycan degradation), as detailed in Table 2.

**Table 2:** KEGG pathways enriched by intersecting DEGs.

ID	Description	*P* value
hsa00511	Other glycan degradation	.04086
hsa04120	Ubiquitin-mediated proteolysis	.04206

DEG = differentially expressed gene, KEGG = Kyoto Encyclopedia of Genes and Genomes.

### 3.4. Identification of characteristic genes for PBC and OP using multiple machine learning methods

**Figure 5.**
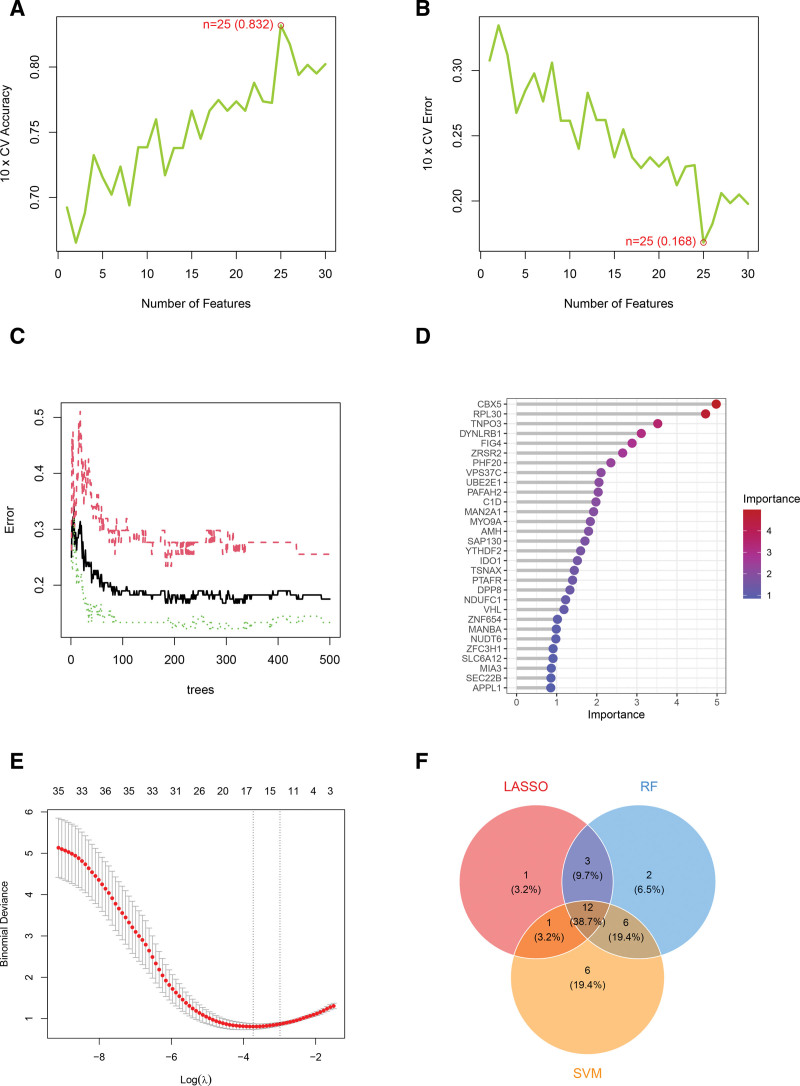
Three machine learning algorithms were used to identify characteristic genes associated with PBC. (A and B) The SVM-RFE algorithm was applied to identify potential diagnostic genes with the highest accuracy and lowest error rates. (C) A random forest analysis was conducted to examine the relationship between the number of trees in the model and the error rate. (D) Diagnostic genes with an importance score >1 were identified using the random forest algorithm. (E) The LASSO algorithm was utilized to determine both the candidate diagnostic genes and the optimal lambda value. (F) A Venn diagram was created to illustrate the overlapping characteristic genes for PBC identified by the LASSO, random forest, and SVM-RFE algorithms. LASSO = least absolute shrinkage and selection operator, PBC = primary biliary cholangitis, SVM-RFE = support vector machine recursive feature elimination.

**Figure 6.**
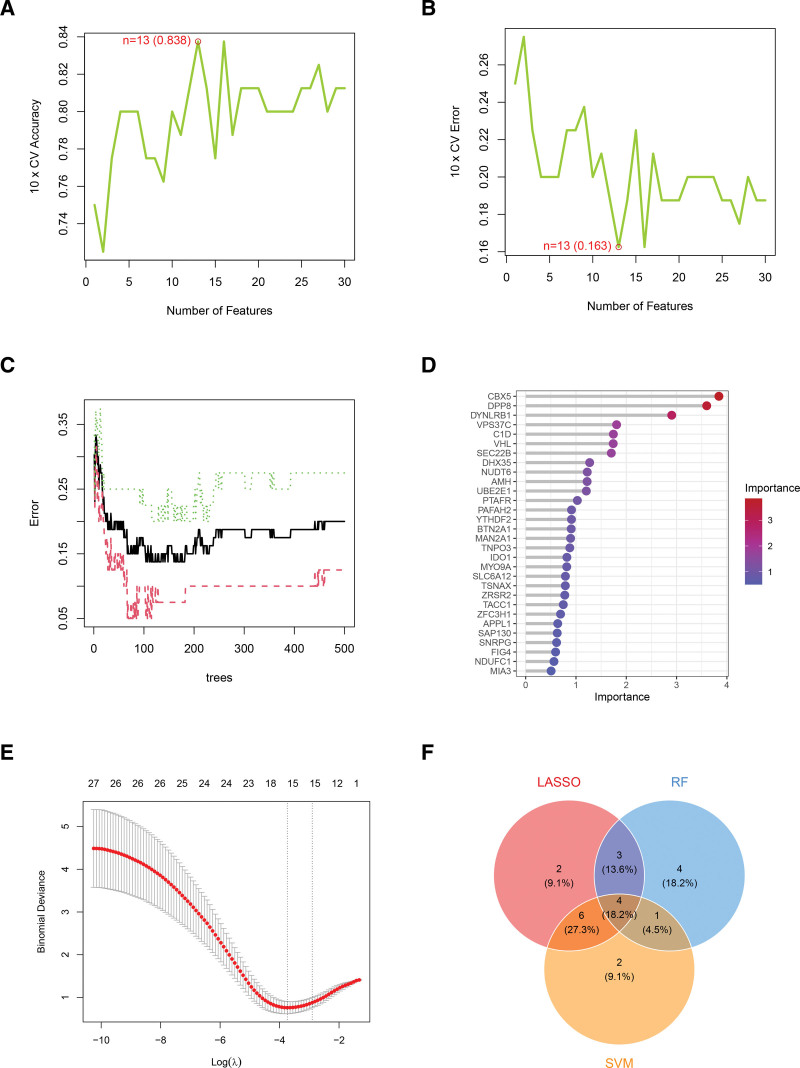
Three machine learning algorithms were used to identify characteristic genes associated with OP. (A and B) The SVM-RFE algorithm was applied to identify potential diagnostic genes with the highest accuracy and lowest error rates. (C) A random forest analysis was conducted to examine the relationship between the number of trees in the model and the error rate. (D) Diagnostic genes with an importance score >1 were identified using the random forest algorithm. (E) The LASSO algorithm was utilized to determine both the candidate diagnostic genes and the optimal lambda value. (F) A Venn diagram was created to illustrate the overlapping characteristic genes for OP identified by the LASSO, random forest, and SVM-RFE algorithms. LASSO = least absolute shrinkage and selection operator, OP = osteoporosis, SVM-RFE = support vector machine recursive feature elimination.

In the samples for PBC, we identified 25 diagnostic genes using the SVM-RFE algorithm. The random forest method revealed 23 diagnostic genes, while 17 diagnostic genes were recognized from intersecting DEGs through the LASSO algorithm. By combining the results of these 3 methods, we discovered 12 overlapping characteristic genes for PBC (Fig. 5). Likewise, as depicted in Figure 6 for OP samples, we selected 13 diagnostic genes using the SVM-RFE algorithm, identified 12 diagnostic genes through the random forest method, and found 15 diagnostic genes with the LASSO algorithm. The combination of these approaches yielded 4 overlapping characteristic genes for OP. The specific names of the disease-specific genes identified by the 3 machine learning methods are detailed in Table 3.

**Table 3:** Intersecting characteristic genes of 3 machine learning methods.

Disease	Characteristic genes			
	LASSO	SVM-RFE	Random forest	Intersecting genes of 3 methods
PBC	*DPP8, TNPO3, PHF20, FIG4, MAN2A1, PTAFR, SLC6A12, ZRSR2, IDO1, VPS37C, PAFAH2, AMH, CBX5, C1D, SEC22B, VHL, TSNAX*	*CBX5, SNRPG, ZRSR2, PHF20, C1D, AMH, RPL30, FIG4, MANBA, MAN2A1, HSPB1, SLC6A12, YTHDF2, SAP130, TSNAX, APPL1, TNPO3, ZFC3H1, MYO9A, NDUFC1, IDO1, DYNLRB1, DHX35, PAFAH2, VPS37C*	*CBX5, RPL30, TNPO3, DYNLRB1, FIG4, ZRSR2, PHF20, VPS37C, UBE2E1, PAFAH2, C1D, MAN2A1, MYO9A, AMH, SAP130, YTHDF2, IDO1, TSNAX, PTAFR, DPP8, NDUFC1, VHL, ZNF654*	*TNPO3, PHF20, FIG4, MAN2A1, ZRSR2, IDO1, VPS37C, PAFAH2, AMH, CBX5, C1D, TSNAX*
OP	*DHX35, PHF20, SAP130, ZRSR2, IDO1, MIA3, VPS37C, AMH, TACC1, CBX5, DYNLRB1, VHL, TSNAX, NUDT6, SNRPG*	*DHX35, ZRSR2, VHL, VPS37C, DYNLRB1, ZNF654, PHF20, SNRPG, TSNAX, MIA3, SEC22B, ZFC3H1, SAP130*	*CBX5, DPP8, DYNLRB1, VPS37C, C1D, VHL, SEC22B, DHX35, NUDT6, AMH, UBE2E1, PTAFR*	*DHX35,VPS37C, DYNLRB1, VHL*

LASSO = least absolute shrinkage and selection operator, OP = osteoporosis, PBC = primary biliary cholangitis, SVM-RFE = support vector machine recursive feature elimination.

### 3.5. Identification of the common diagnostic gene between PBC and OP, validation of ROC curves, and construction of the PPI network

**Figure 7.**
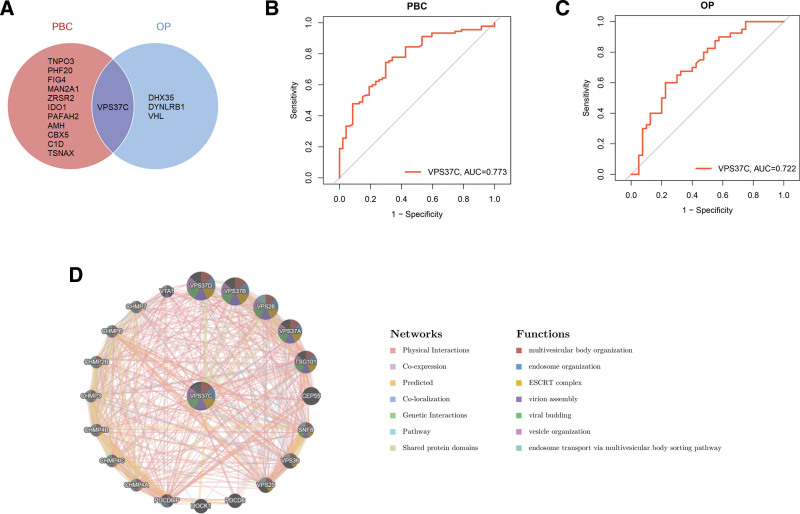
Identification of the common diagnostic gene between PBC and OP. (A) The diagnostic gene shared between PBC and OP. (B) ROC curve for VPS37C in PBC dataset. (C) ROC curve for VPS37C in OP dataset. (D) PPI network associated with VPS37C. OP = osteoporosis, PBC = primary biliary cholangitis, PPI = protein-protein interaction, ROC = receiver operating characteristic, VPS37C = vacuolar protein sorting 37 homolog C.

By analyzing the characteristic genes associated with PBC and OP, we identified a gene that is common to both diseases: vacuolar protein sorting 37 homolog C (VPS37C; Fig. 7A). In the predictive models for each disease, VPS37C showed strong diagnostic potential with an AUC of 0.773 for PBC and 0.722 for OP, both of which exceed the threshold of 0.7. This indicates that VPS37C has significant diagnostic, predictive, and discriminatory power for both conditions (Fig. 7B, C). Further analysis of the VPS37C PPI network revealed that this gene mainly interacts with members of the vacuolar protein sorting and charged multivesicular body protein families. The physiological and pathological processes involved include the organization of multivesicular bodies, endosomes, the endosomal sorting complex required for transport (ESCRT) complex, virion assembly, viral budding, and vesicle organization (Fig. 7D).

### 3.6. Development of ceRNA regulatory networks for the shared diagnostic gene

**Figure 8.**
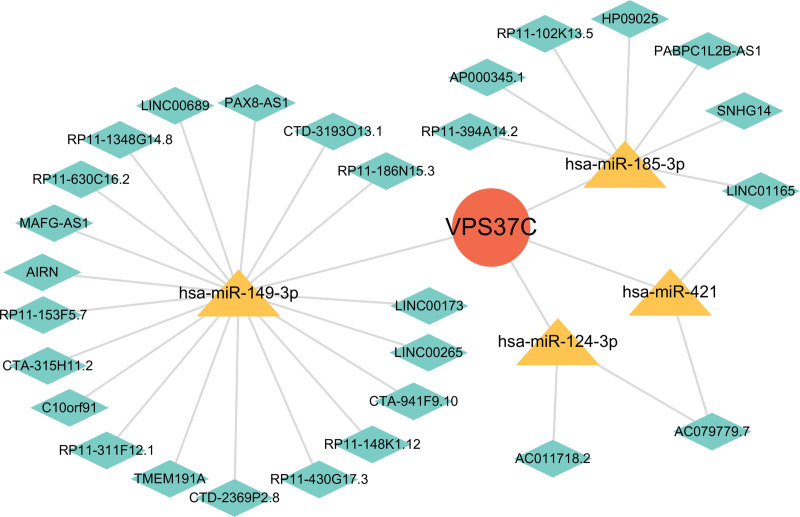
The ceRNA network of VPS37C. ceRNA = competing endogenous ribonucleic acid, VPS37C = vacuolar protein sorting 37 homolog C.

We constructed an lncRNA-miRNA-mRNA ceRNA network to explore the upstream regulatory mechanisms affecting VPS37C dysregulation in both diseases. By integrating data from the miRDB, miRTarBase, and TargetScan databases, we identified miRNAs that can target VPS37C. Next, we utilized ENCORI to identify lncRNAs associated with these miRNAs, ultimately developing the ceRNA network for VPS37C (Fig. 8). This network includes several RNAs involved in immune regulation and immune-related functions, such as miR-149-3p, miR-124-3p, C10orf91, and AIRN, which deserve further investigation.

### 3.7. Analysis of the relationship between the shared diagnostic gene and infiltrating immune cells

**Figure 9.**
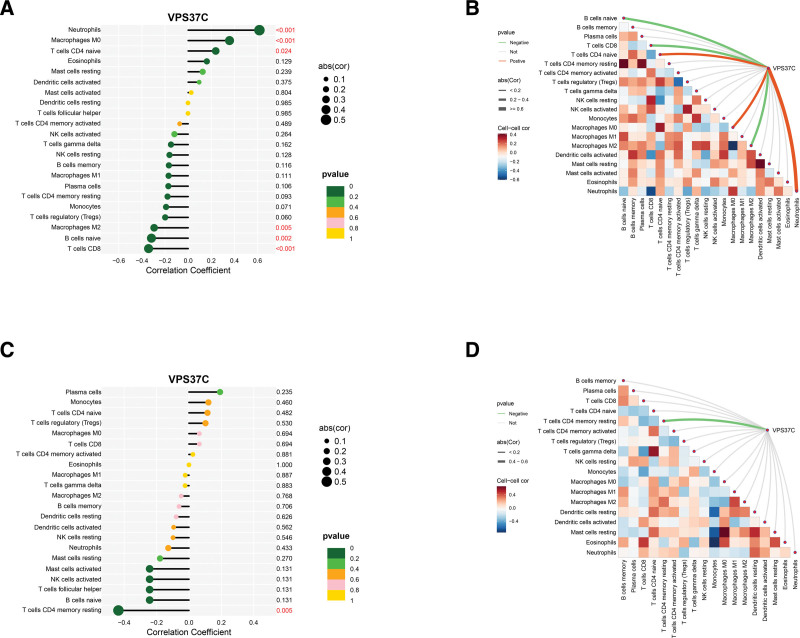
Correlation maps of immune cell infiltration. (A) Relationship between VPS37C and immune infiltrating cells in PBC samples. (B) Correlation between immune infiltrating cells in PBC samples. (C) Relationship between VPS37C and immune infiltrating cells in OP samples. (D) Correlation between immune infiltrating cells in OP samples. OP = osteoporosis, PBC = primary biliary cholangitis, VPS37C = vacuolar protein sorting 37 homolog C.

Using the CIBERSORT algorithm, we examined the correlation between VPS37C and 22 types of infiltrating immune cells. As illustrated in Figure 9, in PBC tissues, VPS37C shows a positive correlation with CD4 naive T cells, M0 macrophages, and neutrophils, while it has a negative correlation with naive B cells, CD8 T cells, and M2 macrophages (Fig. 9A). In tissues from OP, VPS37C exhibits an inverse correlation with CD4 memory resting T cells (Fig. 9C). In addition, in PBC tissues, the resting state of CD4 T cell memory showed a significant positive correlation with the naive state of B cells. Conversely, the M2 macrophages displayed a significant negative correlation with the expression of M0 macrophages (Fig. 9B). In OP tissues, the resting state of mast cells demonstrated a strong positive correlation with M0 macrophages, while eosinophils exhibited a significant negative correlation with monocytes (Fig. 9D). These findings suggest that variations in immune cell infiltration may provide potential targets for therapeutic intervention for both PBC and OP.

## 4. Discussion

PBC is an autoimmune liver disease that causes the destruction of intrahepatic bile ducts. Over time, this condition is often associated with abnormal bone metabolism. OP is one of the most common extrahepatic complications of PBC, significantly increasing the risk of fractures and negatively impacting patients’ quality of life.^[7,8]^ However, the specific molecular mechanisms linking PBC and OP remain poorly understood, and there is a lack of systematic analysis of the regulatory networks involved. This study is the first to utilize bioinformatics and machine learning techniques to identify characteristic genes shared by PBC and OP. This approach offers new insights into the molecular connections between liver health and bone health, paving the way for the development of diagnostic and intervention targets that address both conditions.

In this study, we first employed a two-sample MR framework to systematically evaluate the causal association between PBC and OP. Based on GWAS summary statistics, we selected SNPs significantly associated with PBC as IVs. Using the IVW method as the primary analysis, supplemented by sensitivity tests, we found a significant positive causal association between genetically predicted PBC onset and OP risk. The results indicated that the occurrence of PBC significantly increases the risk of developing OP, with no significant bias detected by the heterogeneity test or the pleiotropy test, confirming the robustness of this causal inference. By establishing this causal relationship, we provide strong support for the association network between “PBC feature genes and OP” that we constructed in this study.

Following the establishment of PBC’s causal effect on OP, we further integrated multi-dimensional bioinformatics strategies to deeply dissect the underlying molecular mechanisms. We first performed transcriptomic analysis by retrieving gene expression profiles of PBC and OP patients and screened the DEGs between disease and control samples. By intersecting DEGs associated with PBC and OP, we identified the common DEGs present in both diseases. The enrichment analysis of these DEGs suggests that they are involved in various BPs, including RNA splicing, ribonucleoprotein complex biogenesis, vacuole organization, and the negative regulation of autophagy. The development of autoimmune disorders is linked to abnormalities in RNA splicing, which can result in structural or expression anomalies of immune-related proteins. This includes the posttranscriptional processing of major histocompatibility complex molecules, leading to abnormal presentation of self-antigens, disrupting immune tolerance, and triggering autoimmune responses against BECs.^[27,28]^ In addition, abnormal synthesis of ribonucleoprotein complexes may hinder the regular expression and function of cytokines and chemokines in immune cells, worsening immune damage to the liver. Vacuoles play a role in the transport and degradation of substances within cells, while autophagy is vital for maintaining cellular homeostasis. In biliary epithelial cells affected by PBC, the improper regulation of autophagy can result in the accumulation of damaged organelles and misfolded proteins that are not cleared efficiently. This buildup can induce endoplasmic reticulum stress in cells, leading to apoptosis or necrosis of biliary epithelial cells. It can also negatively impact the immune microenvironment of the liver, promoting the onset and persistence of inflammation.^[29–31]^ The KEGG enrichment analysis indicates that the intersecting DEGs are primarily concentrated in the ubiquitin-mediated protein hydrolysis pathway. This pathway is essential for maintaining intracellular protein homeostasis. Abnormalities in this pathway can lead to the accumulation of misfolded and damaged proteins that cannot be properly degraded. Such dysfunction may also influence the degradation of immune-related proteins, resulting in the abnormal activation of self-reactive T cells, the survival of BECs, increased cell apoptosis, and exacerbated liver damage in PBC.^[32–35]^

Afterwards, we employed multiple machine learning methods to identify disease-specific genes for PBC and OP. By intersecting these genes, we ultimately identified VPS37C as a gene significantly associated with the pathogenesis of both diseases. VPS37C is located on chromosome 11. It acts as a core subunit of the endosomal sorting complex known as ESCRT-I, forming a trimer with TSG101 and VPS28. This complex participates in various BPs mediated by the ESCRT system.^[36]^ Through synergistic interactions with ESCRT-0, II, and III, as well as Vps4, the ESCRT complex performs essential functions, including membrane remodeling and cleavage, vesicle sorting (such as multivesicular body formation and exosome release), membrane damage repair, and immune regulation. Specific roles of the ESCRT complex include terminating STING signaling, sorting T-cell receptors to halt their signaling, and negatively regulating antigen cross-presentation in dendritic cells.^[37,38]^ In addition, studies have shown that ESCRT proteins help tumor cells evade immune destruction by facilitating the repair of cell membranes. When cytotoxic T lymphocytes release perforin, it forms pores in the membranes of tumor cells. The influx of extracellular Ca^2+^ triggers the rapid recruitment of the ESCRT complex to the damaged area, where it repairs the pore through budding and shedding. This action blocks granzyme entry into the cytoplasm, preventing the intracellular granzyme activity from reaching the apoptotic threshold and thus reducing the apoptosis rates of tumor cells. Experiments have confirmed that knocking out Chmp4b or inhibiting VPS4A function increases the sensitivity of tumor cells to cytotoxic T lymphocyte killing and combined perforin-granzyme treatment. This mechanism offers a novel target for cancer immunotherapy and offers immunological insights for the treatment of PBC and OP.^[39]^

VPS37C may play a significant role in the pathogenesis of PBC through various critical pathways, including membrane repair, vesicle sorting, and regulation of autophagy. The core pathology of PBC at the level of BECs involves the apoptosis of these cells and chronic inflammation. The membrane repair function of the VPS37C-ESCRT system can help counteract damage to the BEC membrane caused by bile acid toxicity and immune attacks. If this system is dysfunctional, it may worsen cell apoptosis and lead to the release of the mitochondrial antigen PDC-E2, which disrupts immune tolerance. Furthermore, at the level of immune regulation, abnormalities in the VPS37C-mediated exosome and microvesicle biosynthesis processes can result in the mis-sorting of self-antigens. These mis-sorted antigens can then be presented to immune cells through extracellular vesicles, triggering the production of antimitochondrial antibodies and activating T-cell responses. On the cellular homeostasis level, disruptions in the macroautophagy and microautophagy pathways involving VPS37C may lead to an abnormal accumulation of proteins within BECs. This accumulation can worsen inflammation and is associated with the senescence-associated secretory phenotype observed in aged BECs in PBC.

In our further studies, ROC analysis demonstrated that VPS37C achieved AUC values exceeding 0.7 for predicting both PBC and OP. Consequently, VPS37C emerges as a diagnostic biomarker with strong predictive capability. Through additional immune infiltration analysis, we found that in PBC tissues, VPS37C positively correlates with CD4 naive T cells, M0 macrophages, and neutrophils, while negatively correlating with naive B cells, CD8 T cells, and M2 macrophages. In OP tissue, VPS37C showed an inverse correlation with CD4 memory resting T cells. Multiple studies have previously suggested that PBC is associated with alterations in immune cell populations.^[40–43]^ These findings indicate that VPS37C may serve as a potential diagnostic biomarker and an immunotherapy target for both PBC and OP.

From a clinical translation perspective, the characteristic gene identified in this study provides potential “dual value.” First, it can serve as an early warning biomarker for OP in patients with PBC. Currently, the clinical assessment of bone metabolic risk in PBC patients mainly relies on bone density testing, which has low sensitivity and does not allow for early prediction. By measuring the expression level of the shared characteristic gene in peripheral blood, a combined predictive model could be developed for precise identification and early intervention in high-risk patients. Second, the gene offers new insights for the development of targeted drugs for “liver-bone co-treatment.” Designing therapeutic interventions that target the characteristic gene and its key regulatory pathways holds promise for simultaneously reducing hepatic inflammation in PBC and improving abnormalities in bone metabolism. This approach could mitigate the potential adverse effects of using a monotherapy on the other system, thereby enhancing the overall efficacy and safety of treatment.

This study has several limitations. Firstly, all research data were sourced from public databases, which may influence the results due to the sample size and the heterogeneity of the population. Moreover, the construction of the machine learning model did not include clinical phenotypic data, such as patient age, disease duration, and treatment regimens. Future research should integrate this clinical information to enhance the model and improve its predictive accuracy. In addition, further testing with clinical samples and cellular experiments is necessary to confirm the expression patterns and functions of the identified characteristic genes.

## 5. Conclusions

This study successfully identified the shared characteristic gene between PBC and OP using interdisciplinary approaches that integrated bioinformatics and machine learning. It provides preliminary insights into the molecular mechanisms underlying their association. By moving beyond traditional single-disease research, this work represents the first systematic exploration of the shared molecular regulatory networks that contribute to the co-occurrence of these conditions. It offers a novel perspective on understanding the interactions between chronic liver disease and OP. Future studies should focus on validating the functional roles of the characteristic gene to clarify its specific mechanisms in PBC-related OP. In addition, integrating clinical data to develop predictive models and intervention strategies could enhance the translation of basic research into clinical applications, ultimately providing new solutions to improve long-term outcomes for patients with PBC.

## Acknowledgments

The authors express their deep gratitude to the GEO database and the MRC IEU database for providing the platform for public datasets.

## Author contributions

**Data curation:** Hongyu Li, Qiaowei Fan.

**Formal analysis:** Hongyu Li, Xinya Guo, Qiaowei Fan.

**Investigation:** Hongyu Li, Qiaowei Fan.

**Methodology:** Hongyu Li, Xinya Guo.

**Software:** Hongyu Li, Xinya Guo.

**Visualization:** Hongyu Li, Qiaowei Fan.

**Writing—original draft:** Hongyu Li, Xinya Guo, Qiaowei Fan.

**Writing—review & editing:** Hongyu Li, Qiaowei Fan.

**Conceptualization:** Qiaowei Fan.

**Funding acquisition:** Qiaowei Fan.

**Supervision:** Qiaowei Fan.








