# Supplementary material for: Mechanisms linking primary biliary cholangitis and osteoporosis: A combined clinical and molecular analysis
Source: Medicine (Baltimore). 2026 May 22;105(21):e48812. doi: 10.1097/MD.0000000000048812 (PMC13200919; doi:10.1097/MD.0000000000048812)
Supplement: Supplementary file 1 [file medi-105-e48812-s001.docx]

**Table S1** The results of Cochran's Q test for assessing heterogeneity.

| **id.exposure** | **id.outcome** | **outcome** | **exposure** | **method** | **Q** | **Q_df** | **Q_pval** |
| --- | --- | --- | --- | --- | --- | --- | --- |
| ebi-a-GCST90061440 | finn-b-M13_OSTEOPOROSIS | OP | PBC | MR Egger | 28.4758230862454 | 33 | 0.69196871849825 |
| ebi-a-GCST90061440 | finn-b-M13_OSTEOPOROSIS | OP | PBC | IVW | 28.4888113183815 | 34 | 0.734406334475103 |

IVW = inverse variance weighted, MR-Egger = Mendelian randomization-Egger, OP = osteoporosis, PBC = primary biliary cholangitis.
