# Supplementary material for: Mechanisms linking primary biliary cholangitis and osteoporosis: A combined clinical and molecular analysis
Source: Medicine (Baltimore). 2026 May 22;105(21):e48812. doi: 10.1097/MD.0000000000048812 (PMC13200919; doi:10.1097/MD.0000000000048812)
Supplement: Supplementary file 2 [file medi-105-e48812-s002.docx]

**Table S3** Detailed information on SNPs serving as IVs in MR analysis.

| SNP | effect_allele.exposure | beta.exposure | beta.outcome | chr | pval.outcome | pval.exposure |
| --- | --- | --- | --- | --- | --- | --- |
| rs11117432 | A | -0.273228 | 0.0263 | 16 | 0.364 | 2.81579e-24 |
| rs1119132 | G | 0.20264 | 0.0189 | 16 | 0.674401 | 6.58097e-10 |
| rs137687 | A | -0.217763 | -0.0049 | 22 | 0.8541 | 2.37411e-23 |
| rs1800693 | C | 0.179759 | 0.0347 | 12 | 0.1863 | 1.19069e-16 |
| rs1808094 | C | -0.127739 | -0.0149 | 18 | 0.5668 | 2.7892e-09 |
| rs2293370 | A | -0.299009 | 0.0263 | 3 | 0.4971 | 5.53988e-25 |
| rs2304256 | A | -0.205245 | 0.0405 | 19 | 0.1748 | 4.42894e-17 |
| rs3131789 | G | 0.180174 | 0.0183 | 6 | 0.4997 | 2.0017e-16 |
| rs34655300 | T | 0.136669 | 0.0296 | 2 | 0.2602 | 5.23227e-10 |
| rs35127065 | T | 0.159067 | 0.0167 | 6 | 0.628699 | 3.82367e-08 |
| rs35350651 | C | -0.191253 | -0.0262 | 12 | 0.3158 | 5.50047e-19 |
| rs35467801 | T | -0.223662 | -0.0305 | 5 | 0.2684 | 6.15319e-19 |
| rs35552227 | T | 0.419016 | 0.0696 | 7 | 0.0543601 | 8.57433e-41 |
| rs3771317 | C | 0.289503 | 0.0243 | 2 | 0.554201 | 2.40215e-22 |
| rs3784099 | A | -0.203016 | -0.0031 | 14 | 0.9134 | 8.30615e-17 |
| rs4780355 | C | -0.199551 | -0.0365 | 16 | 0.1958 | 1.00832e-16 |
| rs4936443 | T | 0.367095 | 0.0213 | 11 | 0.5428 | 5.39262e-35 |
| rs589446 | T | -0.353093 | 0.0294 | 3 | 0.2715 | 1.96381e-55 |
| rs59643720 | C | 0.316412 | 0.0048 | 14 | 0.876 | 2.73275e-38 |
| rs60600003 | G | 0.253264 | 0.0818 | 7 | 0.0586098 | 4.70327e-13 |
| rs6550965 | A | 0.163094 | 0.0177 | 3 | 0.4976 | 3.65258e-14 |
| rs6679356 | T | -0.439362 | -0.0767 | 1 | 0.0797995 | 6.61455e-63 |
| rs7097397 | A | -0.143772 | 0.0234 | 10 | 0.3835 | 2.41958e-10 |
| rs7130339 | A | 0.121645 | -0.0085 | 11 | 0.7446 | 4.66316e-08 |
| rs72699866 | A | -0.195158 | -0.0337 | 14 | 0.368 | 2.88935e-11 |
| rs7674640 | T | 0.216421 | 0.011 | 4 | 0.673301 | 1.56351e-22 |
| rs7805218 | A | 0.128538 | 0.0376 | 7 | 0.1616 | 4.11728e-08 |
| rs79577483 | G | 0.211743 | -0.0247 | 16 | 0.4834 | 1.22999e-11 |
| rs8067378 | G | 0.259633 | 0.0398 | 17 | 0.128 | 1.75186e-33 |
| rs859767 | G | -0.139305 | 0.0045 | 2 | 0.8711 | 1.54323e-09 |
| rs867436 | T | 0.134167 | -0.0196 | 1 | 0.471 | 2.98573e-09 |
| rs928976 | T | 0.410549 | -0.0095 | 6 | 0.7579 | 1.50349e-74 |
| rs9533122 | G | 0.155125 | 0.0062 | 13 | 0.8156 | 5.83042e-13 |
| rs9591325 | C | -0.451884 | -0.0712 | 13 | 0.1855 | 2.14289e-19 |
| rs9652601 | A | -0.239954 | 0.0158 | 16 | 0.5757 | 6.69268e-24 |

SNP = single nucleotide polymorphism, IV = instrumental variable, MR = Mendelian randomization.
