# Supplementary material for: Mechanisms linking primary biliary cholangitis and osteoporosis: A combined clinical and molecular analysis
Source: Medicine (Baltimore). 2026 May 22;105(21):e48812. doi: 10.1097/MD.0000000000048812 (PMC13200919; doi:10.1097/MD.0000000000048812)
Supplement: Supplementary file 3 [file medi-105-e48812-s003.docx]

**Table S4** The 36 intersecting DEGs shared between PBC and OP.

| **Intersecting DEGs** | **Type** | **Intersecting DEGs** | **Type** | **Intersecting DEGs** | **Type** |
| --- | --- | --- | --- | --- | --- |
| TNPO3 | Up-regulated | MANBA | Up-regulated | RPL30 | Down-regulated |
| PHF20 | Up-regulated | PAFAH2 | Up-regulated | NDUFC1 | Down-regulated |
| TMBIM6 | Up-regulated | AMH | Up-regulated | C1D | Down-regulated |
| VPS37C | Up-regulated | IDO1 | Up-regulated | SNRPG | Down-regulated |
| FIG4 | Up-regulated | ZNF654 | Up-regulated | UBE2E1 | Down-regulated |
| DPP8 | Up-regulated | SLC6A12 | Up-regulated | HSPB1 | Down-regulated |
| MIA3 | Up-regulated | BTN2A1 | Up-regulated | NUDT6 | Down-regulated |
| ZRSR2 | Up-regulated | MYO9A | Up-regulated | YTHDF2 | Down-regulated |
| MAN2A1 | Up-regulated | ZFC3H1 | Up-regulated | TSNAX | Down-regulated |
| PTAFR | Up-regulated | DHX35 | Up-regulated | APPL1 | Down-regulated |
| SAP130 | Up-regulated | DYNLRB1 | Down-regulated | VHL | Down-regulated |
| TACC1 | Up-regulated | CBX5 | Down-regulated | SEC22B | Down-regulated |

DEG = differentially expressed gene, OP = osteoporosis, PBC = primary biliary cholangitis.
