# Supplementary material for: Mechanisms linking primary biliary cholangitis and osteoporosis: A combined clinical and molecular analysis
Source: Medicine (Baltimore). 2026 May 22;105(21):e48812. doi: 10.1097/MD.0000000000048812 (PMC13200919; doi:10.1097/MD.0000000000048812)
Supplement: Supplementary file 4 [file medi-105-e48812-s004.docx]

**Table S2** The results of Egger intercept test for assessing pleiotropy.

| **id.exposure** | **id.outcome** | **outcome** | **exposure** | **egger_intercept** | **se** | ***p*-value** |
| --- | --- | --- | --- | --- | --- | --- |
| ebi-a-GCST90061440 | finn-b-M13_OSTEOPOROSIS | OP | PBC | 0.00159784785445913 | 0.0140203999559264 | 0.909955364262789 |

OP = osteoporosis, PBC = primary biliary cholangitis.
